# A Successful Vaginal Hysterectomy for Carcinoma Uteri

**Published:** 1889-01

**Authors:** Wm. H. Wathen

**Affiliations:** Louisville, Professor of Obstetrics and Diseases of Women, Kentucky School of Medicine; Fellow of the American Association of Obstetricians and Gynecologists, etc.; 405 West Chestnut Street


### Buffalo Medical and Surgical Journal

Vol. XXVIII. JANUARY, 1889. No. 6

### Original Communications.

### A SUCCESSFUL VAGINAL HYSTERECTOMY FOR CARCINOMA UTERI.

By WM. H. WATHEN, M. D., of Louisville,

Professor of Obstetrics and Diseases of Women, Kentucky School of Medicine; Fellow of the American Association of Obstetricians and Gynecologists, etc.

[Abstract of a paper read before the Southern Surgical and Gynecological Society at Birmingham, Alabama, December 5, 1888.]

I had written a paper on "Hysterectomy for Malignant Diseases of the Uterus," to read before the Southern Surgical and Gynecological Society in September, but after the meeting was deferred to December, I read the paper before the American Association of Obstetricians and Gynecologists at the meeting of the Congress of Physicians and Surgeons in Washington. I again find my name in the new programme to report upon the same subject. It would not be courteous to the members of this society for me to read that paper again, for an extended abstract of it has been published in many of the medical journals in different sections of the country.

But that I may in a degree fulfil my promise, I have concluded to report a successful vaginal hysterectomy which I did in October, and to make some remarks upon the improved technique of the operation, especially upon that part of the details relating to hemostasis. I operated in the forenoon of October 9th, at the Norton Infirmary, Louisville, on Mrs. B. A., thirty-four years old, of Irish descent, and a mother of five children. When she consulted me, about the first of August, she was suffering with nearly constant bleeding, the blood being mixed with an offensive matter. Her digestive and assimilative functions were not good, and she was badly nourished; she was losing flesh rapidly, and her general appearance indicated approaching cachexia.

I concluded, from the history she gave me of her trouble, that the disease began from twelve to eighteen months before I saw her. . Upon examination, I found carcinoma of the cervix uteri extending up the endometrium, but not involving the vagina or any of the uterine adnexa. The uterus was in normal position and perfectly movable, and no enlargement of pelvic or other glands could be detected. I did not believe all the disease could be removed except by total extirpation of the uterus, and advised her to have the operation done as soon as I could succeed in temporarily improving her local and general condition. She lost but little more blood, and by the first of October looked and felt much better, and there was hardly any further extension of the cancer.

She was prepared for the operation by being well purged, carefully bathed, and the vagina washed out with two gallons of hot water. The hair was cut from her pubes, the parts washed well with ether and a 1-2000 solution of bichloride of mercury. The instruments, sponges, etc., were prepared with all the aseptic care that should govern us in doing successful abdominal surgery. I was assisted in the operation by Drs. H. H. Grant, J. B. Marvin, J. M. Mathews, H. Orendorf, and F. C. Simpson. The water was boiled, and the instruments and sponges were put by the nurse in weak carbolic acid solution. Chloroform was administered, and the operation done after the following fashion. The woman was put in the exaggerated lithotomy position, and the neck of the uterus exposed by a Sims' speculum and retractors, and drawn to the vulva with a heavy volsellum forceps. The vagina was cut away from the cervix at a distance of about one-fourth of an inch from its attachment, and two or three small bleeding arteries secured by catch forceps. Further dissections posteriorly and anteriorly were made with the finger. The pouch of Douglas was first opened and all posterior attachment of the uterus rapidly separated; then the uterus was carefully dissected from the bladder, great caution being observed to prevent wounding this organ or the ureters. Finally, all that held the uterus in position had been divided except the folds of the broad ligaments. The index finger was now hooked well over the left ligament, and it was secured at a distance from the uterus by a catch forceps of my device, which I here show you. The right ligament was clamped in the same way. Both ligaments were then divided with the scissors near the clamps, and the uterus, ovaries, and tubes were pulled away through the vulva.

The uterus was not inverted, and was removed in just twenty minutes. To prevent the possibility of hemorrhage, all bleeding surfaces or points were secured in catch forceps, so that when the operation was done, eight pairs were left in the vagina. She did not lose more than one or two ounces of blood during the operation, and none after it. The small forceps were removed in twenty-eight hours, and the two large ones clamping the broad ligaments were removed in fifty-two hours. A small pledget of sublimated cotton was introduced into the vagina, to hold the forceps apart and to aid drainage, and the vulva was well covered with absorbent cotton, and a T bandage applied. No sutures were used to control hemorrhage or to unite surfaces, and the vaginal vault was left open. The cotton was removed from the vagina when the small forceps were taken off, and it was subsequently used only as a dressing over the vulva. The discharge of necrosed matter, which had been destroyed by the forceps, was rather profuse and offensive for a few days, but after one week it had nearly ceased, and was not offensive in odor. No vaginal washes were used, but the dressing was removed twice daily, and the external parts carefully cleansed. She was allowed to lie on her back or sides, as she preferred, and her water was drawn for one week. Her bowels were moved on the sixth day with sulphate of magnesia, and moved every day or every second day afterward. She had beef peptonoids, beef tea, and mutton broth for three or four days; then she began to take milk and a little solid food, each day increasing the quantity, and after the eighth day she took house diet. She suffered two days from the presence of the forceps, and for two days more from an irritability of the bladder. She was given, during this suffering, one-sixth of a grain of morphine two or three times daily. Her pulse, after the operation and during the first day, was sixty beats per minute. It then ranged from sixty to ninety, seldom getting above seventy-five. Her temperature reached nearly 101° on the second day, probably caused by the local annoyance and pain from the clamps. It then ranged from 98° to 100°. At no time was there any shock or sepsis, and she made an uninterrupted recovery. She was out of bed on the fifteenth day, and left the Infirmary on the nineteenth day.

The vaginal vault had perfectly united at the end of one week. I have examined her several times since she left the Infirmary, and can detect no evidence of any return of the disease. Her general condition and appearance, and all her functions, had improved about twenty-five per cent. at the end of the second week, and she has continued to get better; in fact, she looks and feels perfectly well, goes out on the street, and is attending to her domestic duties.

I interdicted sexual intercourse for three months, but probably this precaution is not entirely necessary, as there has been neither pain nor tenderness on pressure since the third week in the vagina, or the pelvic or abdominal cavities.

A careful microscopical examination of the specimen removed was made by Mr. Simon Flexner, who reports as follows:

LOUISVILLE, Ky., Nov. 3, 1888.

*My Dear Doctor:*

Herewith I beg leave to submit a report on the examination of a specimen handed to me. The specimen consisted of uterus, tubes, and ovaries just removed. The uterus had suffered marked change in configuration. The cervix was changed most, and was the seat of evident degenerative change.

The degenerative process could be traced by the unaided eye through the cervix and about one-half the length of the fundus, where the tissue had a more healthy appearance. This limitation of degenerative process was subsequently confirmed by microscopical examination, as will appear. The tubes were apparently in a healthy condition, and the ovaries presented no abnormal features, save a few cysts.

On microscopical examination, the growth involving the cervix proved to be adeno-carcinoma; as before indicated, the new process extending into the body of the uterus, disappearing about its center. In the mucous membrane of this portion there is considerable hyperplasia of the normally present spindle cells, and foci of round cells are occasionally observable. The glandular structure, however, appeared quite normal. Microscopical examinations of one ovary, at the point where it is attached to the tube, shows no degenerative change. Some connective tissue proliferation had taken place. Beyond this I could observe no change.

The tubes were not examined microscopically.

Pieces from which sections were made were immediately removed and hardened in alcohol.

Very truly, SIMON FLEXNER.

Asepsis, or perfect surgical cleanliness, should be enforced in every detail of the operation. Weak solutions of disinfectants may be used, but I doubt their efficacy, and strong solutions are positively poisonous. I believe the success of vaginal hysterectomy depends largely upon absolute surgical cleanliness, rapidity in operating, and a perfect hemostasis. When the operation is prolonged, and the woman is kept for one or more hours under the influence of an anesthetic, or loses much blood, she is in relatively greater danger of death from shock or sepsis. By the use of clamps to control all hemorrhage, the technique of the operation is so much simplified and improved, that the uterus, etc., can be removed in from ten to twenty minutes, and the loss of blood is no longer an important factor. The clamps also afford an excellent means of drainage, and do away with the necessity of a drainage-tube. But if we follow the technique of Schroeder, Martin, and others, and use sutures to control hemorrhage, and to unite the vaginal and peritoneal surfaces, or to close the vaginal vault, it will require from one to two hours to complete the operation, and the hemostasis is not so perfect. Results have shown that it is best not to close the vaginal opening; and experience has demonstrated that the supposed dangers resulting from intestinal or omental protrusion in the vagina, are mostly imaginary; at least, they are reduced to a minimum.

I believe that the mortality in vaginal hysterectomy can be reduced as low as that in ovariotomy, but I beg to repeat what I have said at another time, "That it is positively criminal for any one to attempt to extirpate a cancerous uterus, or to do pelvic or abdominal surgery, until he learns the anatomy, physiology and pathology of the pelvic and abdominal structures, and knows how to make a correct diagnosis where it is possible to do so; he should also know the general principles and the details of the most approved technique for such operations." Nor should the uterus be removed if there is any evidence of cancerous cachexia, or if, upon a careful physical examination, any structure outside of the uterus in the pelvic cavity is found to be infected. A microscopical examination of a part of the removed tissue by an experienced microscopist and pathologist may aid us very much in diagnosticating cancer of the uterus in its incipiency, when we may expect the best immediate and subsequent results from vaginal hysterectomy.

405 West Chestnut Street.

(This is believed to be the first vaginal hysterectomy for cancer south of the Ohio river.—Ed.)

